# Dynamic patterns of expression for genes regulating cytokinin metabolism and signaling during rice inflorescence development

**DOI:** 10.1371/journal.pone.0176060

**Published:** 2017-04-18

**Authors:** Maria V. Yamburenko, Joseph J. Kieber, G. Eric Schaller

**Affiliations:** 1 Department of Biological Sciences, Dartmouth College, Hanover, New Hampshire, United States of America; 2 Department of Biology, University of North Carolina, Chapel Hill, North Carolina, United States of America; Jawaharlal Nehru University, INDIA

## Abstract

Inflorescence development in cereals, including such important crops as rice, maize, and wheat, directly affects grain number and size and is a key determinant of yield. Cytokinin regulates meristem size and activity and, as a result, has profound effects on inflorescence development and architecture. To clarify the role of cytokinin action in inflorescence development, we used the NanoString nCounter system to analyze gene expression in the early stages of rice panicle development, focusing on 67 genes involved in cytokinin biosynthesis, degradation, and signaling. Results point toward key members of these gene families involved in panicle development and indicate that the expression of many genes involved in cytokinin action differs between the panicle and vegetative tissues. Dynamic patterns of gene expression suggest that subnetworks mediate cytokinin action during different stages of panicle development. The variation of expression during panicle development is greater among genes encoding proteins involved in cytokinin metabolism and negative regulators of the pathway than for the genes in the primary response pathway. These results provide insight into the expression patterns of genes involved in cytokinin action during inflorescence development in a crop of agricultural importance, with relevance to similar processes in other monocots. The identification of subnetworks of genes expressed at different stages of early panicle development suggests that manipulation of their expression could have substantial effects on inflorescence architecture.

## Introduction

Inflorescence development in cereals, including such important crops as rice, maize, and wheat, directly affects the number and size of seeds and is a key determinant of yield [1–3]. The final architecture of the inflorescence is established early in reproductive development based on meristematic activities that establish the branching pattern as well as the positioning of flowers. The rice inflorescence (panicle) consists of a central stem (rachis) with several primary and secondary branches [4, 5]. The primary and secondary branches produce spikelets, each of which produces a single floret. The rice panicle architecture is established when the shoot apical meristem gives rise to a reproductive meristem that produces the inflorescence, which ultimately is the source of the grain. The branches are produced by the primary and secondary branch meristems, the spikelets by spikelet meristems, and these then differentiate into the floral organs.

Cytokinin is a key regulator of meristem size and activity and, as a result, has profound effects on inflorescence development and architecture [1, 6, 7]. Cytokinin levels are controlled primarily through its metabolism, with the basic pathways for its biosynthesis and degradation having been elucidated [8–12]. The biosynthesis of cytokinin involves three key enzymes: isopentenyltransferase (IPT), cytochrome P450 enzyme CYP735A, and LONELY GUY (LOG) cytokinin riboside 5' monophosphate phosphoribohydrolase. These act in sequence to add a prenyl group to the *N*^6^ position of ADP/ATP, hydroxylate the isoprenoid side chain, and then activate the cytokinin by converting it to the free base form [8–10]. In contrast, cytokinin oxidase (CKX) degrades the active cytokinins, lowering their cellular level [8, 12]. The cytokinin response in a tissue is also dependent on its sensitivity to the hormone, as determined by the relative expression of the elements that compose its signal transduction pathway. The initial pathway for cytokinin signal transduction is a multi-step phosphorelay that incorporates cytokinin receptors (HKs), histidine-containing phosphotransfer proteins (AHPs), and type-B response regulators (RRs) [8, 13–18]. These relay the cytokinin signal from the membrane to the nucleus, where the type-B RRs function as transcription factors to regulate gene expression. Type-A RRs are among the targets whose expression is induced by the type-B RRs, and these function as negative feedback regulators for the cytokinin response [8, 19]. In rice, as in most plants, these signaling elements are encoded by multi-gene families, although lineage-specific expansion is found for many of these gene families based on phylogenetic analyses comparing monocots to dicots [13–15, 17]. Interestingly, rice has two classes of receptors. The major class, encoded by four genes, has the cytokinin-binding CHASE domain fused to a histidine kinase domain. In addition, rice has another putative cytokinin receptor in which the CHASE domain is fused to a Ser/Thr kinase domain (CRL4/CHARK, referred to hereafter as CRL4), a receptor structure not found in Arabidopsis or other dicots [16, 20].

Several genetic studies support the role of cytokinins in regulating development of the rice inflorescence. Disruption of the *LOG* gene, involved in cytokinin biosynthesis, results in a failure to maintain meristematic cells in inflorescence meristems, and hence a smaller panicle [11, 21, 22]. Second, through QTL analysis, it was found that the higher yield of the *indica* rice varieties compared to *japonica* was due to reduced expression of the cytokinin oxidase gene *OsCKX2*, a decrease in cytokinin degradation thereby resulting in a larger panicle [23, 24]. More recently, a gene encoding an F-box protein (*LARGER PANICLE*) was identified in rice that, when mutated, results in larger panicles, likely due to decreased expression of *CKX2* [25]. Thus, an increase in cytokinin levels results in an increase in reproductive organs, and consequently an increase in grain yield. Similarly, RNAi of rice *AHP*s results in the production of smaller panicles with reduced seed set, consistent with expression of cytokinin signaling pathway elements also affecting development of the rice inflorescence [26].

Several transcriptome studies have been performed that focused on early stages of rice panicle development. In an initial study 357 genes were identified by cDNA microarray analysis that were expressed differentially during the reproductive stages compared to the vegetative phase [4]. In a subsequent study various developmental stages of rice, including those of panicle development, were analyzed for gene expression using the Affymetrix microarray platform [27, 28]. In a recent study, laser microdissection of meristems from four stages of early panicle development was used for expression profiling by RNA-Seq [29]. These studies indicate that gene expression is dynamically regulated during early panicle development, the RNA-Seq study providing clear evidence that this is also the case for genes related to cytokinin action.

These prior studies are limited due the number of stages of panicle development examined and/or the sensitivity of the experimental approach, which only allowed for a subset of genes related to cytokinin action to be detected with significance. Here we address the regulation of cytokinin action during the early stages of rice panicle development by characterizing gene expression using the NanoString nCounter system [13, 30, 31], which allows for greater sensitivity and more accurate assessment of transcriptional changes than the methods previously employed. Results from our study identify significant differences in abundance as well as dynamic patterns in expression based on 67 probes targeted against genes involved in cytokinin biosynthesis, degradation, and signaling. These results also suggest that gene subnetworks mediate cytokinin action during different stages of panicle development, pointing to how manipulation of their expression can have differential effects on panicle architecture.

## Materials and methods

### Plant materials and growth conditions

*Oryza sativa* ssp. *Japonica* cv. *Nipponbare* seeds were grown in soil (1:1 Pro-mix BX Mycorrhizae and Profile Porous Ceramic Greens Grade) supplemented with water-soluble NPK fertilizer (20-20-20; 2.9 g/L) and Fe fertilizer (Sprint 330; 0.48 g/L) in a green house maintained at 50–80% relative humidity at 30°C during the day and 25°C at night, using a 10 h light/14 h dark cycle.

### Isolation of panicles during early stages of development

Plants were examined daily for panicle promotion by dissection and observation under a stereomicroscope (Leica MZ16) once tillering began. SAMs and early panicles were dissected as described in Furutani et al. (2006) [4]. Specimens were collected into RNAlater (Qiagen) solution and stored at 4°C for up to one week. Each sample consisting of 15–20 dissected SAMs or early panicles was placed in a 1.5-mL microfuge tube with a 3 mm metal bead, frozen in liquid nitrogen, and stored at -70°C until processed for RNA extraction.

### RNA isolation

Frozen panicle samples were ground with a tissue homogenizer (Mixer Mill 400, Retsch) with a 3 mm metal bead. Total RNA was extracted with the E.Z.N.A.^®^ Plant RNA Kit (Omega Bio-tek) according to the manufacturer’s instructions but modified in that we used half the volume of resuspension buffer RB (250μl) due to the small sample weight. The RNA was eluted with 50 μl of DEPC-treated water, with final RNA concentration varying from 20 to 300 ng/μL.

### NanoString analysis

RNA samples for six panicle developmental stages in biological triplicate were directly hybridized with gene-specific color-coded probes and data acquisition carried out with the nCounter Digital Analyzer as described by the manufacturer (NanoString Technologies). The NanoString Codeset was designed and synthesized by NanoString Technologies and is the same used previously to analyze cytokinin function during vegetative development [13]. Normalization and analysis of NanoString data was performed using nSolver Analysis Software 3.0 (NanoString Technologies), making use of six positive-control and eight negative-control probes to generate a standard curve for normalization, as well as five rice-specific reference genes (*EF1*, *ACTIN1*, *ACTIN2*, *UBQ1*, and *ACTIN7*) that spanned a range of counts for CodeSet content normalization. Negative background subtraction, positive control normalization and CodeSet normalization were done using geometric means of the corresponding controls. Clustering was performed by the bottom-up approach of hierarchical agglomerative clustering using a Euclidian distance metric with the distance between two clusters calculated as the mean distance between their elements (Average Linkage method), as provided with the software. Z-score transformation (centering and scaling) was employed for the heat maps to assist in visualizing expression changes. For comparison of panicle gene expression to that found in vegetative tissues, the original vegetative tissue data was reanalyzed using the same methods used for analysis of panicle tissue. All the reaction counts were within the linear dynamic range of the standard curve and all probes detected expression above the background cutoff. Raw data for the NanoString analysis is found in S1 Table. Statistical analyses were performed by using ANOVA with the Holm post-test (http://astatsa.com/OneWay_Anova_with_TukeyHSD/).

### Quantitative Real-Time PCR (qRT-PCR)

cDNA synthesis was performed with the iScript cDNA Synthesis Kit (BioRad), DNase treatment with DNA-free^™^ DNA Removal Kit (Ambion), and qRT-PCR with qPCR iTaq^™^ Universal SYBR^®^ Green Supermix (BioRad), according to the manufacturer’s instructions. qRT-PCR reactions were performed in biological triplicate with technical replicates on the CFX384 Real-Time System (BioRad). *ACT1* was used as the control for qRT-PCR [32]. The ΔΔCt method was used to calculate relative gene expression (RQ) [33]. Primers used for qRT-PCR were: ACT1 (5’-GGTATTGTGTTGGACTCTGG-3’ and 5’-CCGTTGTGGTGAATGAGTAA-3’), LAX1 (5’-CCATCCACTACGTCAAGTTCCT-3’ and 5’-GTTCAGCTCAAGGGCCAGAT-3’), FZP (5’-CACCAACTTCGTCTACACCCA-3’ and 5’-GTGACCGTACGAGCCAATGT-3’), MADS3 (5’-GATGAACATGATGACCGATCT-3’ and 5’-GTGTTCTCGATCCGCTTTAT-3’), LOG (5’-AACTGGTCGAGAGGGGCATA-3’ and 5’-GGGCATCAAGGATTTCGGGA-3’), and RR11 (5’-TCCTCGGAGAATGTGCCAAC-3’ and 5’-GTAGCACGCGGCTGAAGA-3’). Statistical analyses were performed by using the Student’s two-sample t-test or by using ANOVA with the Holm post-test (http://astatsa.com/OneWay_Anova_with_TukeyHSD/) as indicated.

## Results

### NanoString analysis of early rice panicle development

**Fig 1:**
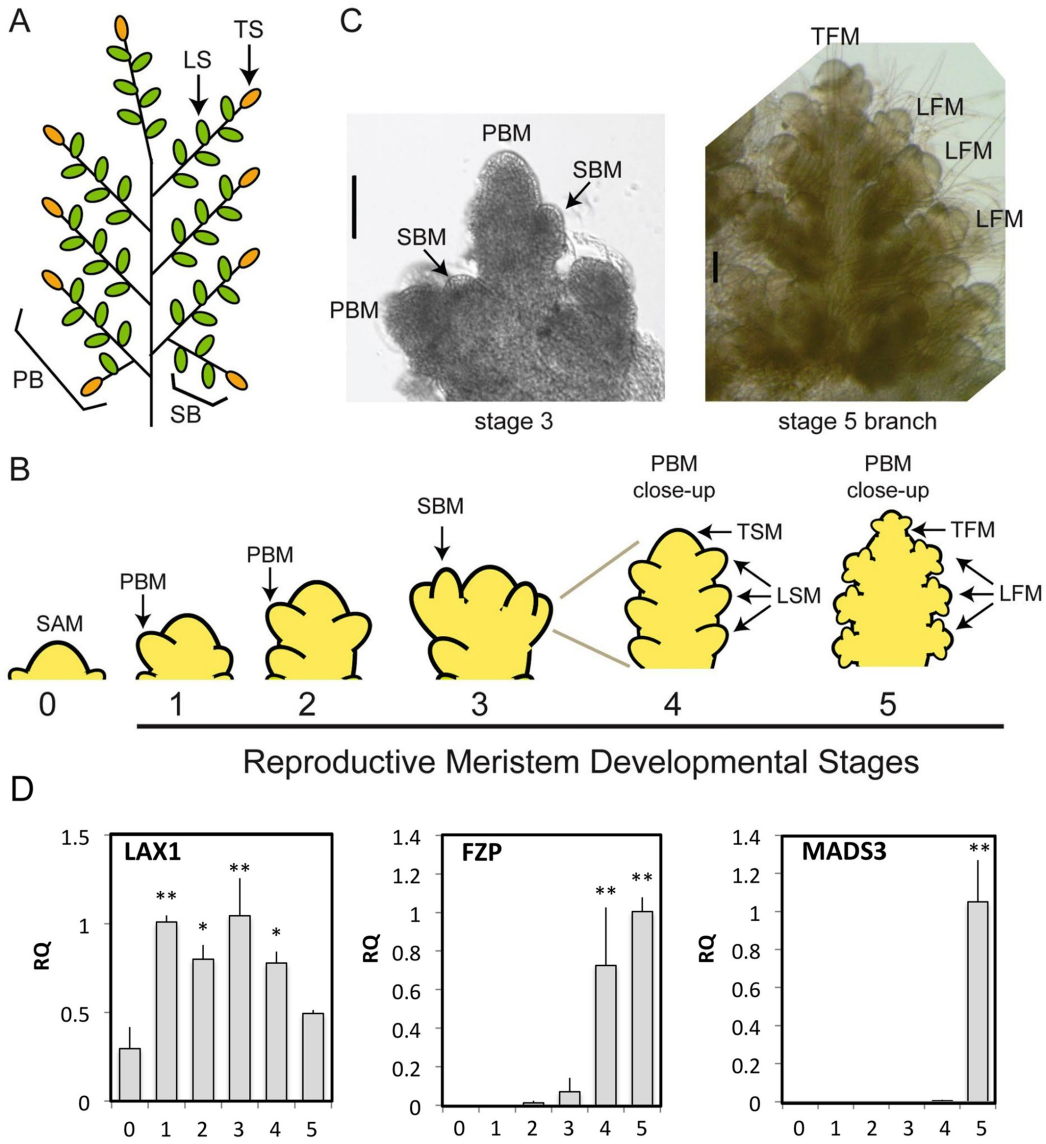
Stages of early panicle development in rice. The rice panicle **(A)** has a defined architecture derived from the meristematic structure established early in inflorescence development **(B, C)**, characterized by diagnostic changes in the gene expression pattern **(D)**. **(A)** Panicle architecture. Representative primary branch (PB), secondary branch (SB), lateral and terminal spikelet (LS and TS) are labeled. **(B)** Developmental stages of the reproductive meristem, which produces primary branch meristems (PBM), secondary branch meristems (SBM), lateral and terminal spikelet meristems (LSM, TSM), which then give rise to floral meristems (LFM, TFM). **(C)** Light microscope images (scale bar = 100μm). **(D)** Diagnostic changes in gene expression occurring during early panicle development based on qRT-PCR using three biological replicates with reproductive stages as defined in **(C)**. Statistical analysis performed by ANOVA with Holm post-test to compare stage 0 to stages 1–5 (* p < 0.05, ** p < 0.01).

To characterize changes in gene expression related to the establishment of the rice panicle architecture, we isolated RNA from the vegetative phase and five early reproductive stages of inflorescence development as in Furutani et al., 2006 (Fig 1A–1C) [4]. Stage 0 (ST0) corresponds to the shoot apical meristem (SAM) just prior to the transition to panicle formation. Stages 1 through 5 are stages of reproductive development for the panicle. In ST1 and ST2, primary branches are progressively initiated; in ST3, formation of secondary branches occurs; and in ST4, spikelet meristems are established, from which floret meristems then emerge in ST5. We examined expression of known panicle-specific genes in the samples to verify accuracy of the sample collection. For this, we performed qRT-PCR using primers for the *LAX1*, *FRIZZY PANICLE* (*FZP*), and *MADS3* genes, which exhibit differential expression during early panicle development (Fig 1D) [4]. Expression of *LAX1*, a gene important for axillary meristem formation [34, 35], is induced following the transition to the reproductive phase and remains elevated throughout the stages of branching and spikelet formation (ST1 through ST4). Expression of the spikelet meristem marker *FZP* is induced during ST4 and ST5 [36], whereas expression of the floral organ identity gene *MADS3* occurs during ST5 with the appearance of stamen and carpel primordia [37].

We examined the regulation of cytokinin function in the developing rice inflorescence by characterizing gene expression using the NanoString nCounter system [30, 31], which allows for greater sensitivity and more accurate assessment than the methods previously used. The NanoString system has a similar sensitivity to that found with quantitative reverse transcription (qRT)-PCR but with the benefit of being able to multiplex probes for many genes at the same time. Furthermore, NanoString analysis uses the RNA transcript sample directly, avoiding bias that can be introduced by cDNA synthesis and amplification, and allowing for quantitative expression comparison of all the genes represented in the probe set. We previously used this methodology to analyze the expression of genes involved in cytokinin biosynthesis, degradation, and signaling in the rice shoot and root [13], and used the same custom probe set for analysis of expression during early panicle development.

**Fig 2:**
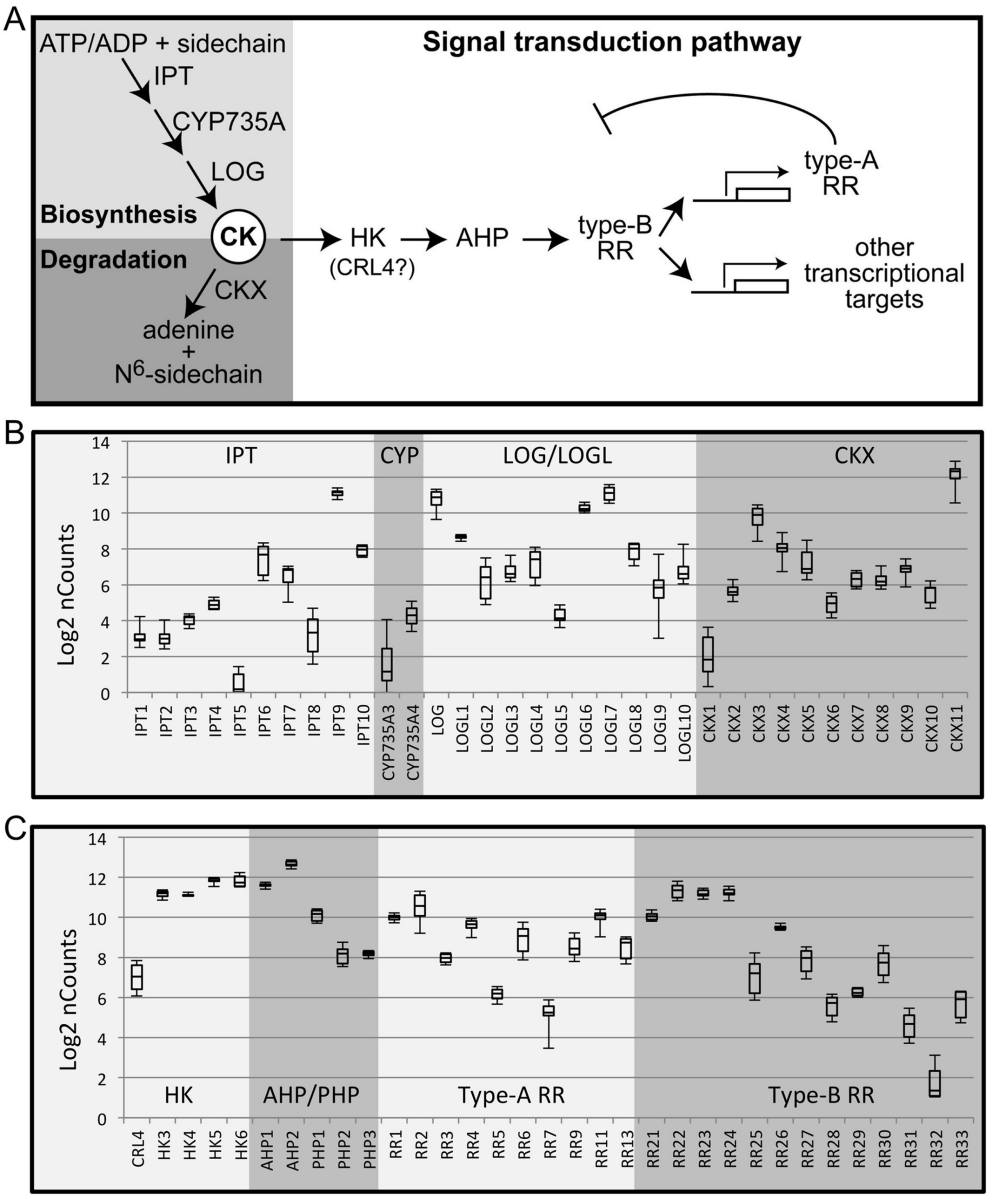
Differing expression levels of cytokinin-related genes in the early rice panicle based on NanoString analysis. **(A)** Genes involved in cytokinin metabolism and signaling. **(B, C)** Box plot analysis for expression of genes involved in cytokinin metabolism **(B)** and signaling **(C)** based on NanoString analysis. Expression was analyzed for stages 0–5 of early panicle development. The bottom and top of each box indicate the 25^th^ and 75^th^ percentile for data expression values, the band in the middle of the box indicates the median expression value, and the ends of the whiskers indicate the minimum and maximum values. Expression values for the box-plot analysis are log_2_ transformed. All probes recognize single genes, except *RR9* which also recognizes *RR10*, and *RR13* which also recognizes *RR8* and *RR12*.

In the NanoString probe set, cytokinin biosynthesis genes are represented by ten isopentenyl transferases (*IPT*s; *IPT1*-*IPT8* involved in cytokinin biosynthesis; *IPT9* and *IPT10* likely serving as tRNA-IPTs), two cytochrome P450 monooxygenases (*CYP735A*), and eleven *LONELY GUY*/*LOG LIKE* (*LOG*/*LOGL*) genes (Fig 2A). There are eleven probes for cytokinin oxidase genes (*CKX*s) encoding cytokinin degradative enzymes. The set also contains probes for genes encoding elements of the cytokinin signal transduction pathway (Fig 2A), including the four cytokinin receptors with histidine-kinase domains (*HK*s), one receptor-like protein with a Ser/Thr kinase domain (*CRL4*), two phosphotransfer proteins (*AHP*s), three pseudo-phosphotransfer proteins (*PHP*s), ten type-A response regulators (type-A *RR*s), and thirteen type-B response regulators (type-B *RR*s) (Fig 2A). Note that there are thirteen type-A *RR*s, but due to high sequence similarity one probe detects both *RR9* and *RR10*, and another probe detects *RR8*, *RR12*, and *RR13*. All other probes are specific for single genes. There are thus 67 probes targeted against 70 genes related to cytokinin action. We evaluated expression of these genes during the vegetative phase (ST0) and five early reproductive stages of inflorescence development (ST1-ST5) (Fig 1A–1C). Positive and negative control probes were used to generate a standard curve, and the data normalized to rice reference genes. All 67 probes related to cytokinin action detected target gene expression above background levels. As described in the following sections, results from the NanoString analysis demonstrate differences in overall expression levels, dynamic changes in expression, and subnetworks of genes based on their expression profiles.

### Differences in expression levels for genes involved in cytokinin metabolism

**Fig 3:**
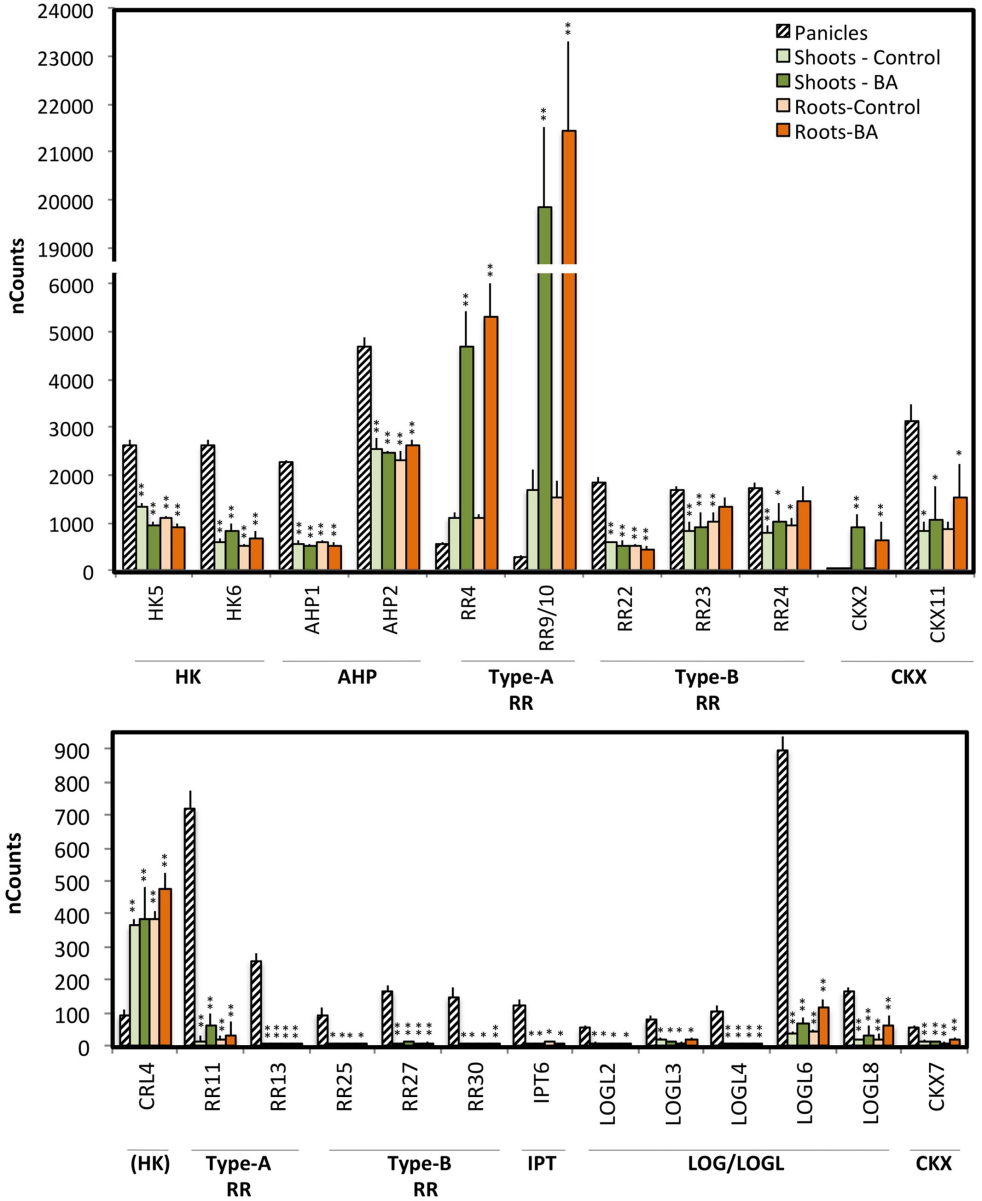
Differential expression of genes involved in cytokinin action between panicles and vegetative tissues. The average gene expression value during early panicle development was compared to that found in rice roots and shoots following treatment for 2 h with 5 μM BA or a vehicle control [13]. Representative genes exhibiting significant differential expression in panicles compared to vegetative tissues are shown (ANOVA with Holm post-test, * p < 0.05, ** p < 0.01).

We used the NanoString dataset to compare expression levels of genes within the gene families, identifying the highly expressed genes that are likely to play a more significant role during panicle development. For this purpose, we generated box plots based on gene expression of cytokinin metabolic genes in stages ST0-5, with the box plots indicating the median and range of expression found during these stages (Fig 2B; S2 Table). Furthermore, to better understand the tissue-specific roles of the genes, we compared the median expression level from early panicles to the expression levels in rice roots and shoots, both basally and treated with the cytokinin benzyladenine (BA) (Fig 3; S1 Fig; S3 Table) [13]. This comparison is enabled because no amplification steps are used during NanoString analysis and the same probe set was used for the earlier study involving rice shoots and roots.

Expression for most of the *IPT* genes (six out of ten) and for the two *CYP735A* genes is low in panicles, but similar to that observed in vegetative tissues (Figs 2B and 3; S1 Fig). *IPT6* and *IPT7* are the most abundant panicle *IPT*s involved in cytokinin biosynthesis. *IPT6* was previously hypothesized to be a pseudogene due to an inability to detect its expression in rice [38], however we find that *IPT6* exhibits substantially higher expression in developing panicles than it does in vegetative tissues (~24-fold higher), accounting for this discrepancy between our study and the earlier study, as well as suggesting a more specific role for *IPT6* in panicle development. We note that the sensitivity of the NanoString technology also allowed for detection of *IPT6* expression above background in root and shoot tissues. *IPT7* is generally the most abundant *IPT* in vegetative tissues, with its expression in the panicle being substantially higher than that in shoots, but comparable to that in roots. The tRNA-IPTs (*IPT9* and *IPT10*) are the most abundant *IPT*s in panicle and vegetative tissues, but are expressed at higher levels in panicles than in vegetative tissues.

*LOG*, *LOGL1*, *LOGL6*, and *LOGL7* of the *LOG/LOGL* family exhibit the highest expression in the early panicles (Fig 2B). *LOGL6* transcripts are particularly enriched (about 25-fold) in panicle compared to vegetative tissues, the other three *LOG/LOGL* genes exhibiting similar transcript levels in panicle and vegetative tissues (Fig 3; S1 Fig). The other less-abundant *LOGL* genes exhibit significant differences in their expression in the early panicles compared to vegetative tissues. Four (*LOGL2*, *LOGL3*, *LOGL4*, and *LOGL8*) are expressed 4 to 20-fold higher in the panicle, whereas one (*LOGL5*) is expressed about 5-fold lower in the panicle. The *log* mutant was originally identified in rice based on a screen for defects in shoot meristems, mutants exhibiting a reduction in panicle size, abnormal branching, and defects in flower development [11]. Loss of an abundant member of the LOG family likely contributes to the mutant defect, the presence of additional abundant family members potentially ameliorating the phenotype to some extent. In general, the *IPT* and *LOG* genes are expressed at higher levels in the early panicle compared to the vegetative tissues, likely due to the significance of cytokinins to meristem maintenance and development which plays a key role in early panicle development [1, 6, 7].

Expression of the genes encoding proteins involved in cytokinin degradation is also high in the early panicle (Fig 2B). *CKX11* exhibits the highest expression level of the CKX family in the panicle as well as in vegetative tissues, but *CKX11* is expressed about three-fold higher in the panicle compared to vegetative tissues (Fig 3). *CKX3* is the next most abundant member of the *CKX* family in the panicle (Fig 2B), its expression being similar to that in the vegetative tissues treated with cytokinin. Interestingly, expression of *CKX2* (which accounts for the QTL associated with yield due to its effects on panicle architecture [23, 24]) is relatively low in the panicle compared to other *CKX* family members (Fig 2B); expression is three-fold higher than basal level but substantially lower than that in cytokinin-treated shoots (Fig 3). Overall the high level of *CKX* expression would complement the high level of expression for biosynthesis genes and would facilitate rapid changes in cytokinin levels as needed for growth and development.

### Differences in expression levels for genes involved in cytokinin signaling

The cytokinin signal transduction pathway involves three families of positive regulatory elements (HKs, AHPs, and type-B RRs) (Fig 2A), and genes for these are highly expressed in early panicles consistent with the importance of cytokinin for the formation of panicle meristems (Figs 2C and 3; S1 Fig; S2 Table). Of the four *HK*s, *HK5* and *HK6* are expressed at the highest level with both being substantially elevated in the early panicle compared to vegetative tissues (Figs 2C and 3; S1 Fig; S3 Table); transcript levels of *HK3* and *HK4* are expressed at similar levels in panicle and vegetative tissue (S1 Fig). *CRL4*, which encodes the putative cytokinin receptor-like protein is expressed at a substantially lower level than the HKs in the panicle, and is also expressed at lower levels compared that found in vegetative tissues (Fig 2C). Both *AHP1* and *AHP2*, which encode the two intermediary phosphotransfer proteins, are also expressed at substantially higher levels in the early panicle as compared to vegetative tissues (Figs 2C and 3). Of the 13 type-B *RR*s, which encode DNA-binding transcription factors that mediate the primary transcriptional response to cytokinin, *RR21*, *RR22*, *RR23*, *RR24*, and *RR26* are the most highly expressed family members in the panicle (Fig 2C). The three most abundant family members (*RR22*, *RR23*, and *RR24*) are all expressed at higher levels in panicles compared to vegetative tissues (Fig 3). In addition, among less abundant type-B *RR*s, *RR25*, *RR27*, and *RR30* are more highly expressed in the panicles compared to vegetative tissue (Fig 3). Overall, expression of genes for the HK receptors, AHP phosphotransfer proteins, and type-B RRs that make up the initial signaling circuit is higher in the early panicle than in vegetative tissues.

Cytokinin signal transduction also involves negative regulatory elements (type-A RRs and PHPs) that can feed back and desensitize the pathway (Fig 2A). The type-A RRs are cytokinin primary response genes whose transcription is rapidly induced in response to the hormone [8, 19, 39, 40]. They are hypothesized to compete with type-B RRs for phosphotransfer from the AHPs, thereby decreasing the level of activated type-B RRs, as well as to potentially desensitize the pathway via interactions with other regulatory targets. There are striking differences between type-A *RR* expression in panicles compared to vegetative tissues, suggesting tissue-specific roles for these genes (Figs 2C and 3). The most abundant type-A *RR*s in the panicle are *RR1*, *RR2*, *RR4*, and *RR11* (Fig 2C). Expression of *RR4* and *RR9/10* is substantially lower in the panicle than the basal expression level in the vegetative tissues (Fig 3), of interest because *RR4* and *RR9/10* are two of the most abundant type-A *RR*s in the vegetative tissues, *RR9/10* being the most abundant [13]. Transcript levels of *RR1* and *RR2* are higher in the panicle than the basal level in the shoots, but lower than that observed following cytokinin treatment (Fig 3). Of particular interest, *RR11* and *RR8/RR12/RR13* are relatively abundant in the panicle but not in the vegetative tissues, being expressed at more than 40-fold higher levels in the panicle (Fig 3), suggesting that these type-A *RR*s may play a particular role in panicle development. The PHPs (pseudo-phosphotransfer proteins) lack a conserved phosphorylated histidine residue found in the AHPs, and interfere with AHP function to down-regulate cytokinin signal transduction [8, 41, 42]; genes for the three *PHP*s of rice are expressed at lower levels in the panicle than the *AHP*s, and similarly to their levels in vegetative tissues (Fig 2C; S1 Fig).

### Dynamic patterns in gene expression during early panicle development

All 67 NanoString probes for cytokinin action detected expression for their targets above background levels, with 65 doing so for all six stages of early panicle development (ST0-5), and the remaining two probes for five of the six stages, thereby providing an accurate assessment of changes in expression throughout this critical developmental process. Interestingly, developmental variation is greater among genes for cytokinin metabolism and the type-A *RR*s than for the genes in the primary signaling pathway (Fig 2B and 2C). This suggests the signal transduction pathway is largely poised for cytokinin perception, and that regulation is primarily through local changes in cytokinin levels coupled with negative feedback from the type-A RRs. For example, when considering the predominant family members making up the primary signaling pathway (four *HK*s, two *AHP*s, and five type-B *RR*s), the maximal fold-change for any of these during panicle development is 2-fold (Fig 2C). In contrast, the genes encoding biosynthetic enzymes vary substantially more: the two *CYP735A*s vary from 3.2 to 16.7-fold, the two predominant cytokinin-biosynthesis *IPT*s from 4.0–4.3-fold, and the four predominant *LOG/LOGL*s from 1.3 to 3.2-fold. Among the cytokinin oxidases, the predominant two genes vary from 4.1 to 5.0-fold, and the *CKX2* associated with seed yield varies 2.3-fold. Among the type-A *RR*s, the most abundant four genes vary from 1.4 to 4.3-fold, including *ARR11* which varies 2.6-fold in the panicle. Also of potential significance is the variation in expression levels found for less abundant members of the genes for cytokinin biosynthesis, degradation, type-A RRs, as well as for the type-B RRs (Fig 2).

**Fig 4:**
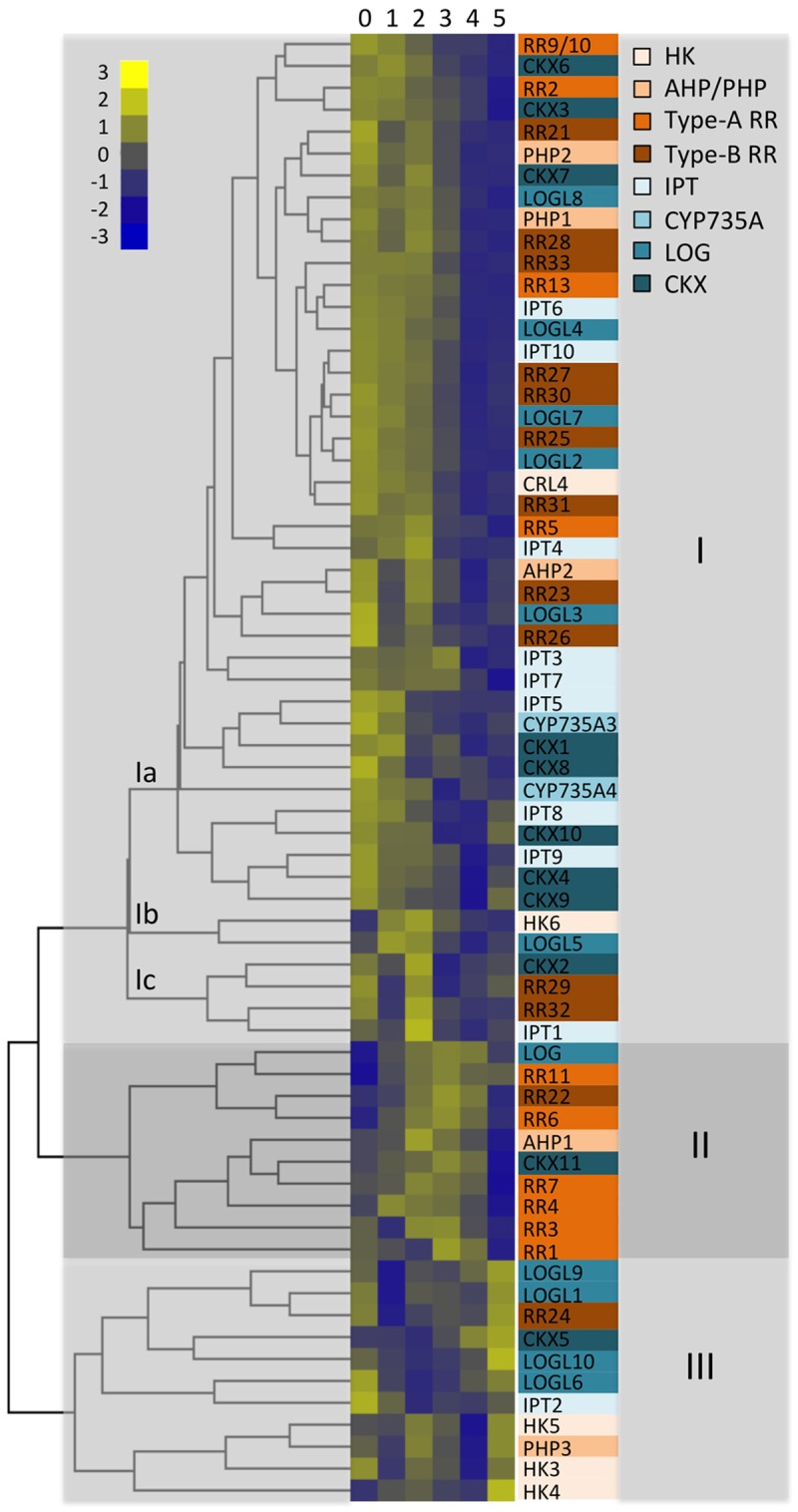
Dynamic changes for expression of genes involved in cytokinin action during early panicle development. A cluster analysis was performed based on Euclidian distance between gene expression at stages 0–5 of early panicle development. This is plotted as a heat map with a dendrogram. Genes are color-coded based on whether they are involved in cytokinin metabolism (in blue) or signaling (in orange), with differing shades for each of the families involved in these processes. Genes fall into three major subfamilies (I, II, and III) based on the cluster analysis.

To reveal differential gene expression during the course of the early panicle development and to find co-regulated genes, we performed a clustering analysis based on Euclidian distance between gene expression at the different stages. This is plotted as a heat map with a dendrogram and color-coded gene families for easier navigation in Fig 4 (heat maps for expression changes found within gene families can be found in S2 Fig). Genes are color-coded based on whether they are involved in cytokinin metabolism (blue) or signaling (orange), with differing shades for each of the families involved in these processes. This cluster analysis supports subnetworks of genes for cytokinin action involved in the establishment of panicle architecture, with three main groups exhibiting peak expression during ST0-ST2 (Group 1), ST2-ST4 (Group II), and ST5 (Group III) stages of panicle meristem development. These groups thus represent subnetworks derived from the total potential network of genes involved in the control of cytokinin action.

**Fig 5:**
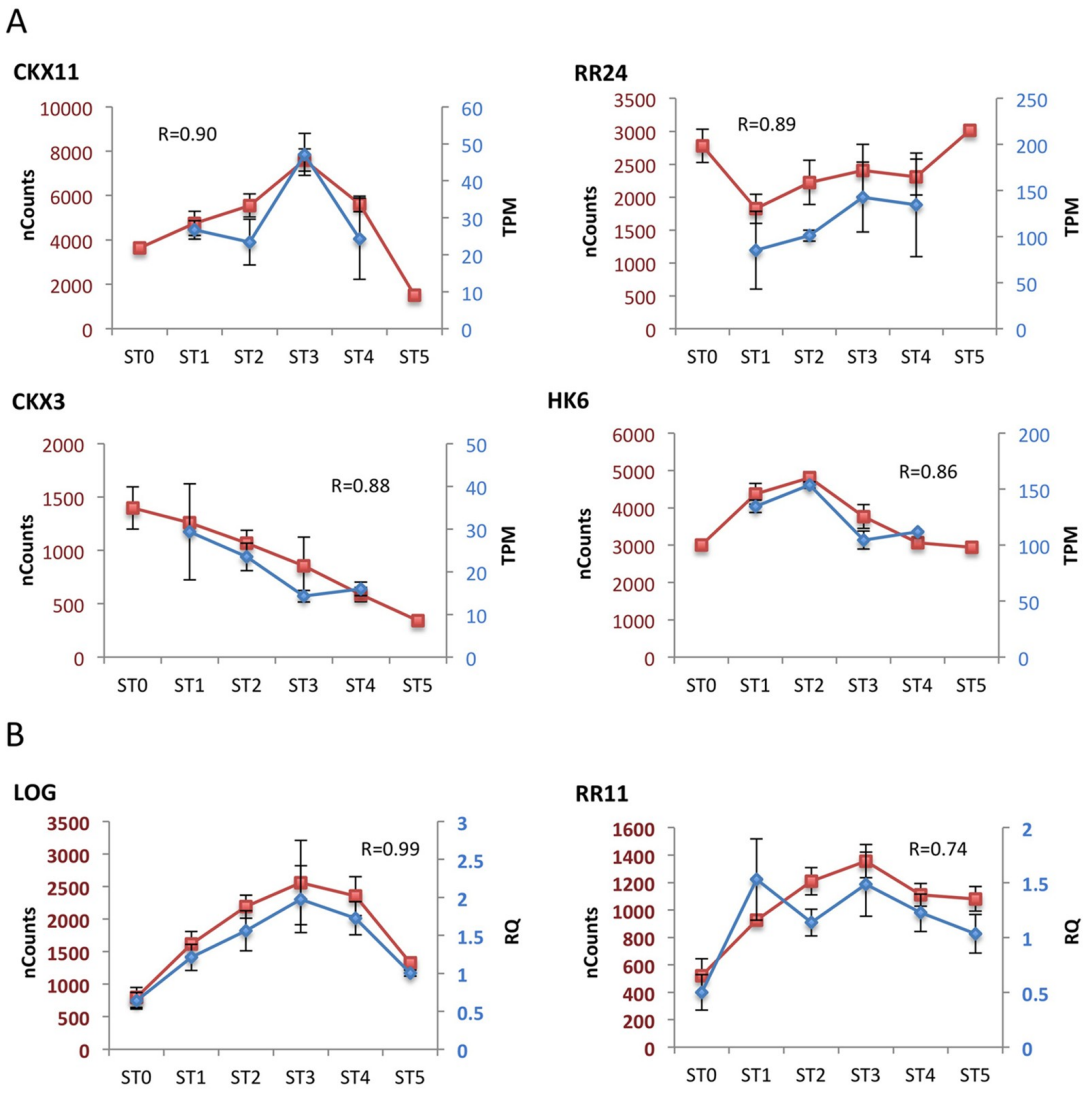
Confirmation of expression changes for representative genes during panicle development. NanoString expression data is compared to expression data derived from RNA-Seq **(A)** or qRT-PCR **(B)**. Genes are from subfamilies I (*CKX3*, *HK6*), II (*CKX11*, *LOG*, *RR11*), and III (*RR24*), based on the cluster analysis (Fig 4). NanoString expression is given in normalized nCounts. The RNA-Seq dataset from panicle meristems [29] covers stages 1 through 4 of early development and is given in transcripts per million (TPM). qRT-PCR data is given as a relative gene expression (RQ) based on an actin control gene.

Group I includes genes that exhibit higher expression in the SAM and during primary branch initiation (from ST0 to ST2), with a decrease in expression during the later stages. This is the largest of the three groups, and is composed of three sub-groups (Ia, Ib, and Ic) (Fig 4). Group I includes the two most abundant *IPT* genes (*IPT6* and *IPT7*), both tRNA-IPT genes (*IPT9* and *IPT10*), and one of the most abundant *LOGL* genes (*LOGL7*), the differences in expression for all of these being significant when compared to later stages of panicle development. *CKX3*, one of the most abundant *CKX*s, as well as *CKX4*, *CKX8*, and *CKX9*, are all significantly enriched members of Group I; the expression changes observed by NanoString with *CKX3* are consistent with a prior RNA-Seq analysis where *CKX3* was detected above background (Fig 5) [29]. Although the more abundant genes for the primary signaling pathway do not exhibit the same variability for expression found with metabolism genes, some are significantly enriched members of group I. This includes the *HK6*, which is found in Subgroup Ic, which is characterized by having maximal expression at ST2 during primary branch meristem formation, an expression pattern we confirmed by qRT-PCR (Fig 5). Group I also includes the type-B *RR*s, *RR21* and *RR25*. Negative regulators of signaling are also found in Group I, which is significantly enriched for two type-A *RR*s (*RR2* and *RR13*) as well as the two most abundant *PHP*s (*PHP1* and *PHP2*).

Group II is characterized by activation of gene expression at ST2 –ST4, stages at which the majority of branch and spikelet meristems are formed. This small group is enriched for the highly expressed *LOG* gene as well as for *CKX11*, the most abundant of the CKX family member in the early panicles, both patterns being confirmed by RNA-Seq (Fig 5A) [29]. Among genes involved in the signaling pathway, Group II is enriched for the abundant type-B *RR*, *RR22*. However what is most striking, given the relatively small size of Group II, is the enrichment for type-A *RR*s, with *RR4*, *RR6*, and *RR11* all being significantly enriched, qRT-PCR results being consistent with the *RR11* enrichment (Fig 5B).

Group III genes are particularly high at ST5 when flower organ meristems emerge, some of these also being activated at ST0 in the SAM. With regards to cytokinin metabolism, Group III is particularly enriched for members of the *LOG* family, both *LOGL9* and *LOGL10* being significantly enriched, as well as for *CKX5*.

The gene for the abundant type-B *RR24* also trends higher during these later stages of development, consistent with prior RNA-Seq analysis (Fig 5) [29].

## Discussion

To clarify the role of cytokinin action in inflorescence development, we used the NanoString nCounter system to analyze gene expression in the early stages of rice panicle development. Results from our study identify significant differences in abundance as well as dynamic changes in expression based on 67 probes targeted against genes involved in cytokinin biosynthesis, degradation, and signaling. These results also suggest that gene subnetworks, derived from the overall network of genes involved in cytokinin action, regulate different stages of panicle development, pointing to how manipulation of their expression can have differential effects on panicle architecture. As discussed below, results from our study complement and extend prior expression and genetic studies that implicate cytokinin action in the control of early panicle development.

### Comparison of NanoString-derived data to prior expression studies

Our gene expression data complement and extend several prior studies focused on early panicle development. In an initial study, 357 genes were identified by cDNA microarray analysis that were expressed differentially during the reproductive stages compared to the vegetative phase [4]. This study employed the same panicle sampling stages that we used (ST0-ST6), allowing us to directly compare results. Two cytokinin-related genes were found to be differentially regulated during the early panicle developmental stages by Furutani et al. (2006) [4]. The transcript level of *CKX5* was enriched at ST4 and ST5, and that of *RR22* was enriched at ST4, consistent with our NanoString analysis (Fig 4).

Recently, a more extensive dataset was acquired by laser microdissection of meristems from four stages of early panicle development with expression profiling by RNA-Seq [29]. Meristem types in the RNA-Seq dataset approximately correspond to the stages from ST1 to ST4. Rachis meristem (RM) corresponds to ST1; primary branch meristem (PBM) corresponds to ST2; elongated primary branch with axillary meristem (ePBM/AM) corresponds to ST3; spikelet meristem (SM) corresponds to ST4. Eighteen of the genes we investigated were above background at the four stages in the RNA-Seq dataset and, even though the micro-dissection focused only on meristematic tissue, 11 of these exhibited a high positive correlation coefficient (R from 0.57 to 0.90) when compared to our NanoString dataset. We plotted four genes *CKX3*, *CKX11*, *RR24*, and *HK6* with particularly high R (higher than 0.86) relative to the NanoString data (Fig 5A). These represent members of Groups I, II, and III, as defined by our cluster analysis, and thus further verify the NanoString data and the dynamic changes in expression we observe during early panicle development.

### Comparison of NanoString-derived data to prior genetic studies

Several genes involved in cytokinin action have been implicated in the control of panicle development based on genetic analysis. These include *LOG* and *CKX2*, which were initially isolated based on their loss-of-function phenotypes [11, 23]. Both of these are involved in cytokinin metabolism, and our data indicate that there is greater expression variability during development for such genes than for genes in the primary signaling pathway. *LOG* is one of the most abundant of the *LOG* family members for cytokinin biosynthesis in the early panicle based on NanoString analysis, and belongs to group II based on cluster analysis, its peak of expression in ST2-4 occurring when the majority of branch as well as spikelet meristems are formed. *In situ* analysis indicates that *LOG* is expressed in SAM, primary and secondary branch meristems, and floral meristems [11]. The NanoString expression analysis is consistent with the *log* mutant phenotype, which is characterized by a severe reduction in panicle size, with decreased branching and decreased flower number [11]. We observe a decrease in *LOG* expression during ST5, when floral organ primordia are formed; *log* mutants develop all types of floral organs but often exhibit a decrease in the number of the inner floral organs. We identified *LOGL6* and *LOGL7* as having similar expression levels to *LOG*, suggesting that these too may play a significant role in regulating panicle development, although their differing dynamics suggests that they may not recapitulate the *LOG* mutant phenotype.

A second gene involved in cytokinin metabolism, *CKX2*, was identified as a major QTL (Gn1a) affecting grain yield [23]. *CKX2* encodes a cytokinin oxidase and its reduced expression results in increased cytokinin levels [23], which correlates with increased grain yield per panicle. This inverse correlation between *CKX2* expression and grain yield has been observed based on natural variation between different rice strains or when CKX2 expression is specifically down-regulated by RNAi or CRISPR-based approaches [23, 43, 44]. Interestingly, NanoString analysis indicates that *CKX2* is of medium abundance in early panicles within the *CKX* family. This may allow for greater variation in its expression level without severely perturbing panicle development and, in this respect, it will be of interest to determine whether mutations in the more abundant *CKX* family members negatively affect panicle development. We find that *CKX2* is a member of group Ic based on cluster analysis, with maximal expression during ST2 a major stage for primary branch formation, and thus the effects of *CKX2* mutations on panicle size and yield are likely related in part to panicle branching.

Effects on panicle development have also been found by taking targeted approaches to modify the expression of cytokinin signaling elements, in particular *AHP*s and type-A *RR*s. The two rice *AHP*s are found in group Ia (*AHP*2) and II (*AHP1*) based on cluster analysis, but there is only limited variation of expression during the panicle stages and thus they can be considered as expressed throughout panicle development. *AHP2* is the most highly expressed of the two family members. The role of the *AHP*s was examined by using RNAi with the *AHP2* sequence, which substantially reduced expression of *AHP2*, the closely related *AHP1*, as well as the *PHP*s to some extent [26]. The mutants exhibited smaller panicles with reduced seed set, although specific aspects of panicle architecture were not quantified. The expectation based on our data is that branch and spikelet formation would be reduced in the mutants compared to wild type.

*RR6*, like many of the type-A *RR*s is a member of group II, and is most highly expressed during the stages when the branch meristems are formed. The type-A RRs are negative regulators and so ectopic overexpression of *RR6*, as examined by Hirose et al. [45], would be predicted to result in a greater level of negative regulation, and with this expanding to other stages of early panicle development. The mutant lines exhibited small panicles with reduced branching, reduced spikelet number, and abnormal flowers that are sterile [45], consistent with defects throughout all stages of early panicle development.

## Conclusion

The results obtained from the NanoString expression analysis provide insight into how genes involved in cytokinin action regulate inflorescence development in a crop of agricultural importance, with relevance to similar processes in other monocots. The expression levels for many genes involved in cytokinin action differ between the panicle and vegetative tissues, suggestive of differing functional roles tailored to the specific needs of these tissues. Substantial differences in expression were identified among gene-family members, and these may point the way toward which genes play predominant roles in early panicle development. The identification of subnetworks of genes expressed at different stages of early panicle development points to how manipulation of their expression could have differential effects on inflorescence architecture.

## Supporting information

S1 Fig NanoString expression comparison of genes involved in cytokinin action between panicles and vegetative tissues. The average gene expression value during early panicle development was compared to that found in rice roots and shoots following treatment for 2 h with 5 μM BA or a vehicle control.
(PDF)

S2 Fig Dynamic changes for expression of gene family members involved in cytokinin action. A cluster analysis was performed based on Euclidian distance between gene expression at stages 0–5 of early panicle development. This is plotted as a heat map with a dendrogram for each family of genes. ^#^ Significant differences based on a T-Test between the two stages with maximum and minimum expression (P < 0.05). *Significant differences when comparing expression across all stages based on ANOVA with Holm post-test (P < 0.05).
(PDF)

S1 Table Raw NanoString expression data for genes involved in cytokinin action. (XLSX)

S2 Table Normalized nCounts for gene expression during stages ST0-ST5 of early panicle development (LOG2 transformed). (XLSX)

S3 Table Comparison of gene expression between panicles, shoots, and roots based on NanoString analysis. (XLSX)
