# Supplementary figures and images for: Dynamic patterns of expression for genes regulating cytokinin metabolism and signaling during rice inflorescence development
Source: PLoS One. 2017 Apr 18;12(4):e0176060. doi: 10.1371/journal.pone.0176060 (PMC5395194; doi:10.1371/journal.pone.0176060)

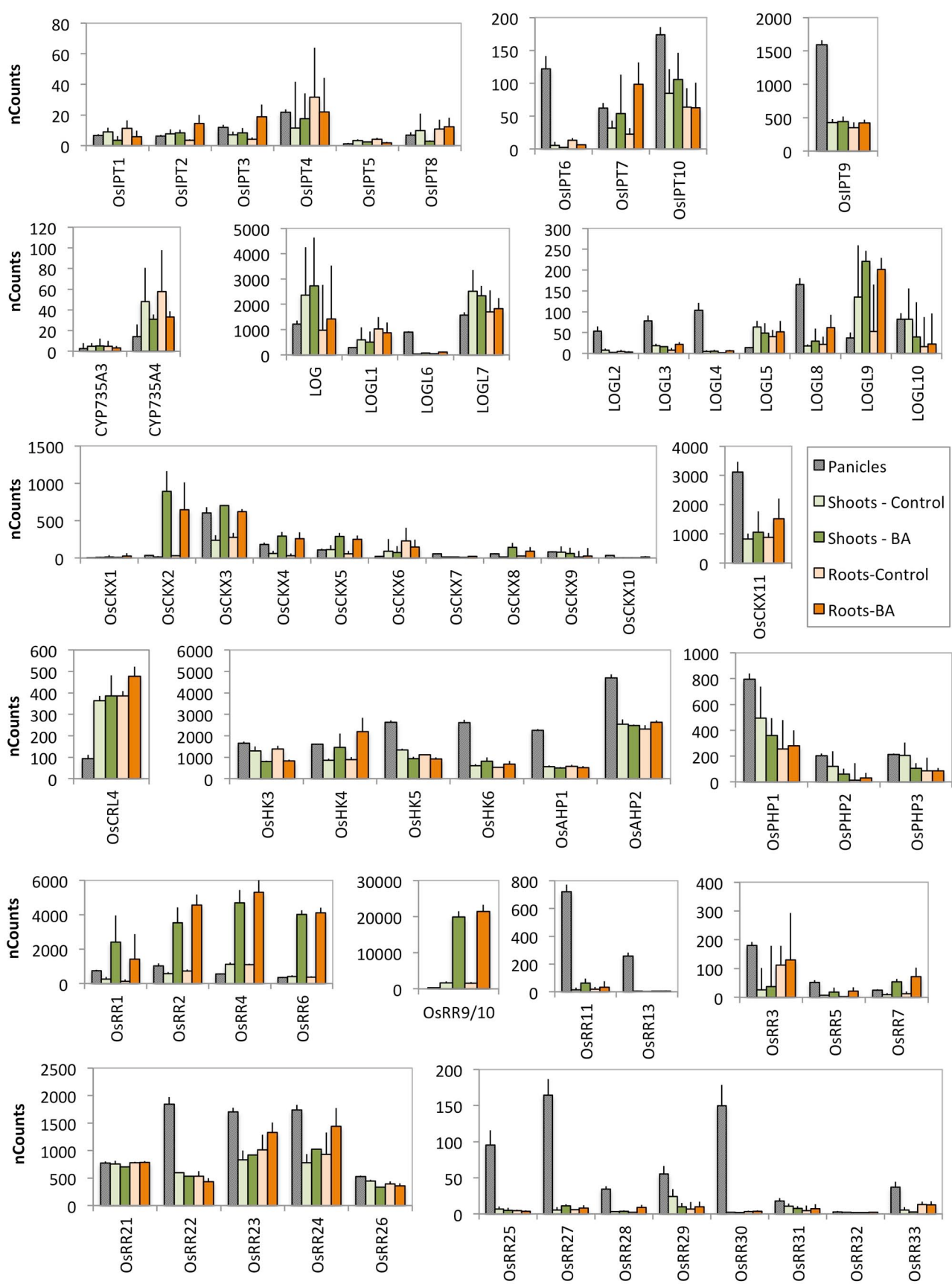

S1 Fig

Supplement: S1 Fig — The average gene expression value during early panicle development was compared to that found in rice roots and shoots following treatment for 2 h with 5 μM BA or a vehicle control. (PDF) [file pone.0176060.s001.pdf]

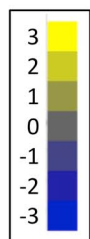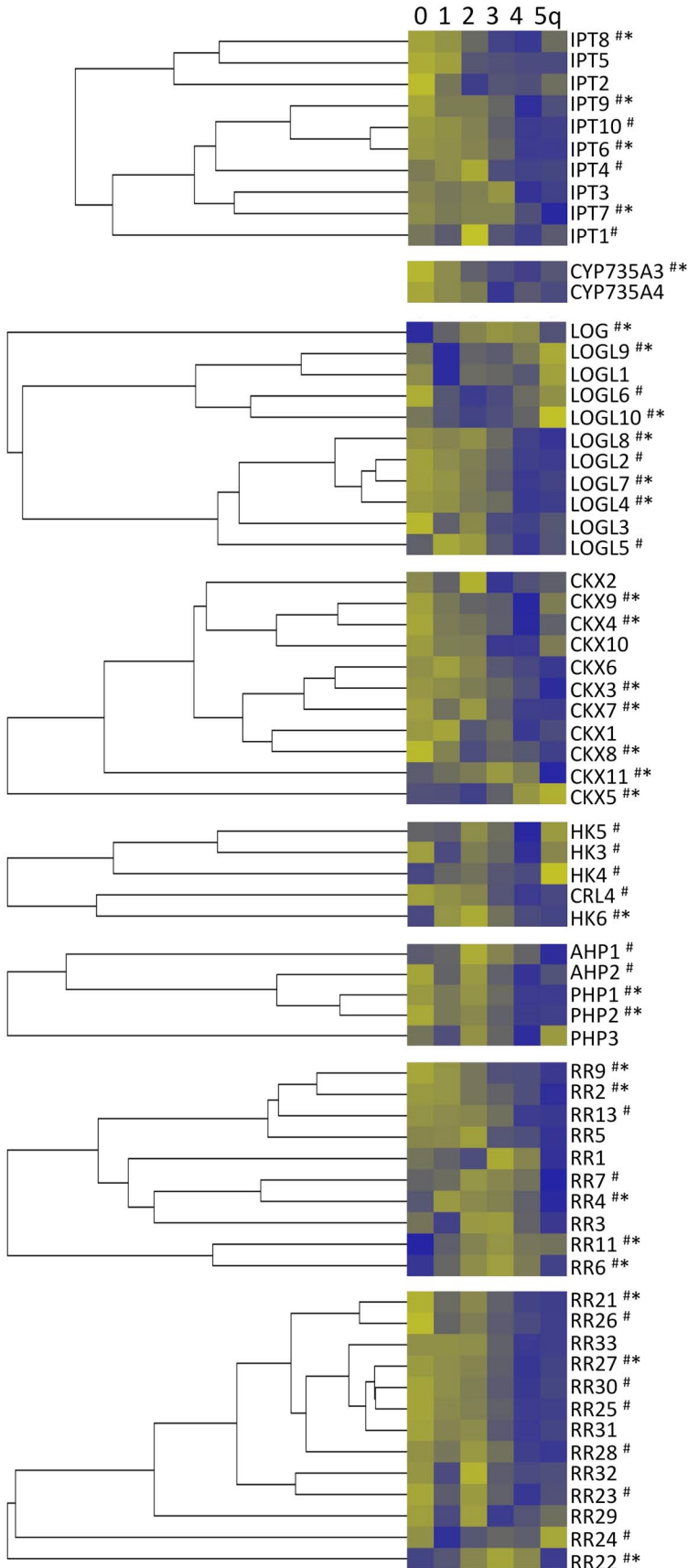

S2 Fig

Supplement: S2 Fig — A cluster analysis was performed based on Euclidian distance between gene expression at stages 0–5 of early panicle development. This is plotted as a heat map with a dendrogram for each family of genes. # Significant differences based on a T-Test between the two stages with maximum and minimum expression (P < 0.05). *Significant differences when comparing expression across all stages based on ANOVA with Holm post-test (P < 0.05). (PDF) [file pone.0176060.s002.pdf]
